# Serum Dickkopf-1 signaling and calcium deposition in aortic valve are significantly related to the presence of concomitant coronary atherosclerosis in patients with symptomatic calcified aortic stenosis

**DOI:** 10.1186/s12967-015-0423-2

**Published:** 2015-02-15

**Authors:** Zuzana Motovska, Teodora Vichova, Magdalena Doktorova, Marek Labos, Marek Maly, Petr Widimsky

**Affiliations:** Cardiocentre, Third Medical Faculty Charles University and University Hospital Kralovske Vinohrady, Prague, Czech Republic; Department, of Radiology, University Hospital Kralovske Vinohrady, Prague, Czech Republic; National Institute of Public Health, Prague, Czech Republic

**Keywords:** Calcified aortic stenosis, Dickkopf-1 signaling, Calcium deposition in aortic valve

## Abstract

**Background:**

The study aimed to assess serum RANKL:OPG ratio, Dkk-1 and deposition of calcium in aortic valve in relation to the presence of concomitant coronary atherosclerosis in patients with symptomatic calcified aortic stenosis (CAS).

**Methods:**

OPG, soluble RANKL and Dkk-1 were measured in 218 consecutive patients who were undergoing cardiac catheterization because of symptomatic CAS. Values of studied compounds were compared between patients without (Group A) and with (Group B) coronary atherosclerosis. Computed tomography derived Agatston score was assessed by using 256-slice CT.

**Results:**

Presence of coronary atherosclerosis was related to significantly (p = 0.007) higher OPG and to significantly (p = 0.004) lower Dkk-1. Coronary atherosclerosis was also associated with a trend towards a decrease of RANKL*.* RANKL/OPG Ratios (mean (95% C.I.)) were: 20.04 (16.58; 24.23) in Group A and 12.69 (9.96; 16.17) in Group B, resp., p = 0.018). After adjustment, the difference in RANKL:OPG ratios was no longer significant. Multivariable regression underscored the significance of difference in Dkk-1 (p_after adjustement_ = 0.020). Group A patients had significantly higher Dkk-1, significantly higher deposition of calcium in aortic valve and were symptomatic in significantly younger age (p < 0.001) as compared to group B patients: Agatston score (mean (95% C.I.)) 4069.9 (3211.8; 5134.5) and 2413.5 (1821.3; 3198.1), p = 0.007.

**Conclusions:**

Dkk-1 and deposition of calcium in aortic valve differ significantly in relation to the presence/absence of coronary atherosclerosis in patients with symptomatic CAS. A positive association was found between Dkk-1 and calcium load in aortic valve in patients with symptomatic CAS and angiographically normal coronary arteries.

## Introduction

Calcified aortic valve disease, a progressive mineralization of aortic valves, is responsible for the most common valve malfunction in adult population – aortic stenosis. Every 50^th^ of individuals ≥ 65 years old has calcified aortic stenosis (CAS), with 80% progressing to symptoms requiring a surgery to prevent death and to improve quality of life [1,2].

It has been recognized, that the most significant predictor of clinical progression of calcified aortic valve disease is the load of calcium in aortic valve [3]. The process of calcium deposition in aortic valve is a multi-factorial event where several pathways interact and influence disease progression [4]. OPG (Osteoprotegerin)/RANKL (Receptor Activator Of Nuclear Factor Kappa B Ligand)/RANK cytokine axis and Wingless tail (Wnt)/Dickkopf-1 (Dkk-1) signaling have been linked to the development of atherosclerosis and might be along the causal pathway in regulation of valvular calcification in CAS. Published observations suggest that RANKL/RANK interaction stimulates vascular/valvular calcification. Osteoprotegerin acts as a decoy receptor for the pro-osteoclastic RANKL, and therefore OPG may inhibit the process of calcification [5]. The OPG:RANKL ratio determines the net effect on osteoclasts. The effects of Dkk-1 on bone are mediated by inhibition of Wnt signaling, which directly impaired new bone formation and limited OPG expression, thereby shifting the OPG:RANKL ratio to favor bone resorption [6].

Degenerative aortic valve disease shares many features with atherosclerosis. The initial plaque of aortic stenosis is similar to that seen in coronary artery disease [7]. However, a significant proportion of patients with severe aortic stenosis have no coronary atherosclerosis. Therefore, the study was designed to asses serum RANKL/OPG ratio, Dkk-1 signaling and deposition of calcium in aortic valve in patients with symptomatic CAS in relation to the presence of concomitant coronary atherosclerosis.

## Methods

The research was carried out according to the principles of the Declaration of Helsinki. Patients gave informed consent and the Ethics committee of University Hospital Kralovske Vinohrady in Prague (Czech Rep.) approved the study.

Study group consisted of 218 consecutive patients who were undergoing cardiac catheterization being considered for aortic valve replacement for symptomatic CAS in a tertiary care institution [8] from 3/2010 – 6/2012. Patients with bicuspid aortic valve were excluded from the participation in this study. The history of chronic kidney disease was an exclusion criterion because causing disorder of bone and mineral metabolism, and extra-skeletal calcifications. Furthermore, every patient who underwent cardiac catheterization was examined on renal function. Patients with GFR <60 mL/min/1.73 m^2^ were excluded. Patients gave informed consent and the local ethics committee approved the study. Baseline characteristics of study population are presented in Table 1.

**Table 1:** **Comparison of baseline characteristics of patients with symptomatic calcified aortic stenosis and without (Group A) or with (Group B) concomitant coronary atherosclerosis**

	**Group A**	**Group B**	**P-value**
	**N = 112**	**N = 106**	
Age years, mean (SD)	69.7 (11.5)	75.7 (8.7)	<0.001
Men N (%)	60 (53.6)	63 (59.4)	0.657
Obesity N (%)	43 (38.7)	34 (32.4)	0.633
Hyperlipidemia N (%)	37 (33.0)	38 (35.9)	0.892
Hypertension N (%)	70 (62.5)	83 (78.3)	0.024
Diabetes mellitus N (%)	33 (29.5)	43 (40.6)	0.172
Smoking (anytime) N (%)	26 (23.4)	41 (39.4)	0.081
IACE therapy N (%)	42 (37.5)	43 (41.0)	0.895
ARB therapy N (%)	12 (10.71)	20 (19.05)	0.170
Statin therapy N (%)	33 (23.7)	51 (48.6)	0.011
Pressure gradient across the aortic valve (mmHg);			
Mean ± SD	44.4 ± 17.9	40.4 ± 15.5	0.128
Median (25^th^ to 75^th^ percentile)	43 (32 to 57)	41 (28 to 50)	
Peak aortic valve velocity (m/s)			
Mean ± SD	4.1 ± 0.8	4.0 ± 0.8	0.107
Median (25^th^ to 75^th^ percentile)	4.2 (3.6 to 4.7)	4.0 (3.4 to 4.4)	
Severe aortic stenosis N (%)	100 (89.3)	85 (18.2)	0.088

SD – standard deviation; IACE – inhibitors of ACE; ARB – angiotensin II receptor blockers.

Blood samples were collected during cardiac catheterization. After standard processing, serum was stored at −70°C until assayed. Samples were assayed with OPG ELISA kit (Biovendor, Laboratorni Medicina, Brno, Czech Rep.), sRANKL ELISA kit (Biovendor, Laboratorni Medicina, Brno, Czech Rep.) and Dkk-1 ELISA kit (Biovendor, Laboratorni Medicina, Brno, Czech Rep.), respectively, according to the manufacturer’s protocol. Limits of detection were: 0.1 pmol/l of OPG, 0.4 pmol/l of sRANKL, and 0.01 ng/ml of Dkk-1.

Values of circulating OPG, sRANKL, Dkk-1 were compared between patients without (Group A) and with (Group B) concomitant coronary atherosclerosis (coronary artery stenosis ≥ 50%). Patients in group A had angiographically normal coronary arteries and without coronary calcification.

**Figure 1:**
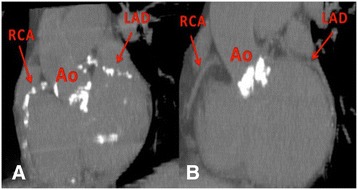
**Demonstration of typical noncontrast CT study of a patient with severe calcified aortic stenosis and extensive difuse coronary calcifications (picture A) in contrast with patient without concomitant coronary disease (picture B).**

Computed tomography (CT) derived Agatston score was assessed by using 256-slice Brilliance iCT (Philips Medical Systems, Best, The Netherlands) (Figure 1). Scans were performed with prospective ECG gating at 75% of RR interval. Standardized protocol for calcium scoring was used (gantry rotation time 270 ms, 120 kv tube voltage and 80 mA tube current). For quantifiaction, the data were transferred to commercially available workstation Brillance Workspace Portal v.2.6.0.27 (Philips Medical Systems, Best, The Netherlands).

### Statistical analysis

Continuous data are presented as arithmetic and geometric means for normally and log-normally distributed variables, respectively, and 95% confidence intervals are used to describe the amount of uncertainty associated with estimate of a population mean. For the analysis, the asymmetrically distributed dependent variables were log-transformed. The comparison of groups was based on Student’s *t*-test and multiple linear regressions, which were used to adjust for potential confounders. Categorical data are presented as absolute frequencies and percentages and were analyzed using Fisher’s exact test and the proportional odds model. A statistical analysis was performed by statistical software Stata, release 9.2 (Stata Corp LP, College Station, TX). All statistical tests were evaluated at a significance level of 0.05.

## Results

Presence of coronary atherosclerosis was related to significantly higher OPG and to significantly lower Dkk-1 in comparison to presence of normal coronary arteries in patients with CAS (Table 2). Concomitant coronary atherosclerosis was also associated with a non-significant trend towards a decrease of sRANKL*.* RANKL:OPG Ratio (mean (95% C.I.)) was 20.04 (16.58; 24.23) in Group A and 12.69 (9.96; 16.17) in Group B, respectively, p = 0.018).

**Table 2:** **Comparison of studied compounds between patients with calcified aortic stenosis and without (Group A) or with (Group B) concomitant coronary atherosclerosis**

Variable	**A (N = 112)**	**B (N = 106)**	**P-value**	**P**_**1**_ **-value**	**P**_**2**_ **-value**	**P**_**3**_ **- value**
OPG (pmol/l);	6.06 (5.58; 6.58)	7.27 (6.74; 7.84	0.007	0.703	0.852	0.933
gmean (95% C.I.)						
sRANKL (pmol/l);	121.43 (103.37; 142.64)	92.24 (73.35; 115.99)	0.238	0.787	0.860	0.977
gmean (95% C.I.)						
Dkk-1 (ng/ml);	1.44 (1.25; 1.66)	1.01 (0.87; 1.17)	0.004	0.010	0.016	0.020
gmean (95% C.I.)						

P-value for comparison A and B.

P_1_-value for comparison A and B after adjustment for age.

P_2_-value for comparison A and B after adjustment for age and diabetes mellitus.

P_3_-value for comparison A and B after adjustment for age, diabetes mellitus, hypertension, hyperlipidemia, and smoking.

OPG: P-value for age < 0.001; P-value for diabetes mellitus = 0.007; P-value for hypertension = 0.949, P-value for hyperlipidemia = 0.219, P-value for smoking = 0.470.

RANKL: P-value for age < 0.001; P-value for diabetes mellitus = 0.131; P-value for hypertension = 0.018, P-value for hyperlipidemia = 0.425, P-value for smoking = 0.467.

OPG : RANKL Ratio: P-value for age < 0.001; P-value for diabetes mellitus = 0.018; P-value for hypertension = 0.019, P-value for hyperlipidemia = 0.244, P-value for smoking = 0.347.

Dkk-1: P-value for age < 0.832; P-value for diabetes mellitus = 0.112; P-value for hypertension = 0.568, P-value for hyperlipidemia = 0.601, P-value for smoking = 0.904.

gmean – geometric mean; C.I. - Confidence Interval.

After adjustment for variables that significantly influenced levels of OPG (age, diabetes mellitus) and RANKL (age, hypertension), the difference in RANKL:OPG ratio between Group A and Group B was no longer significant (Table 2). Conversely, multivariable regression underscored the significance of difference in Dkk-1 signaling in relation to the presence/absence of concomitant coronary atherosclerosis in patients with CAS (p_after adjustement_ = 0.020).

**Figure 2:**
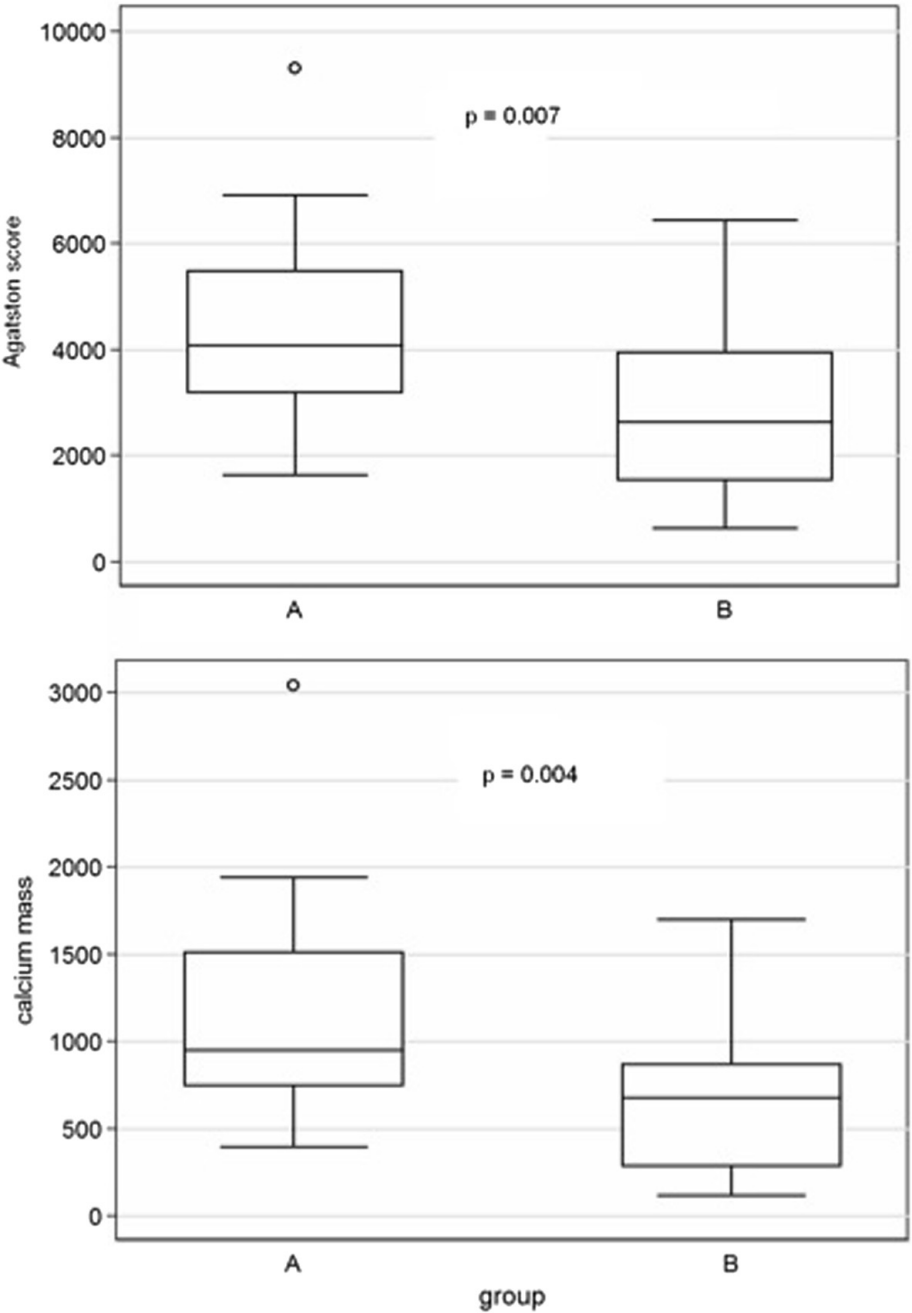
**Calcium deposition in aortic valve in patients with calcified aortic stenosis in relation to the presence of concomitant coronary atherosclerosis.** Group **A** (N = 112) – patients with CAS and without coronary atherosclerosis, Group **B** (N = 106) – patients with CAS and with coronary atherosclerosis.

Patients with CAS and without coronary atherosclerosis had significantly higher deposition of calcium in aortic valve and were symptomatic in significantly younger age as compared to patients with CAS and with concomitant coronary atherosclerosis (Table 1, Figure 2): Agatston score (mean (95% C.I.)) 4069.9 (3211.8; 5134.5) and 2413.5 (1821.3; 3198.1), p = 0.007.

## Discussion

Active osteochondrogenic differentiation and signaling were demonstrated in aortic valves in CAS [9]. Load of calcium in valve tissue was recognized as the substantial sign of disease progression. Recent studies have led to an understanding of the roles of cytokine and cell-signaling pathways involving OPG/RANKL/RANK and Wnt in disease pathogenesis. The cross talk between the two systems seems to be of biological relevance [10,11].

**Figure 3:**
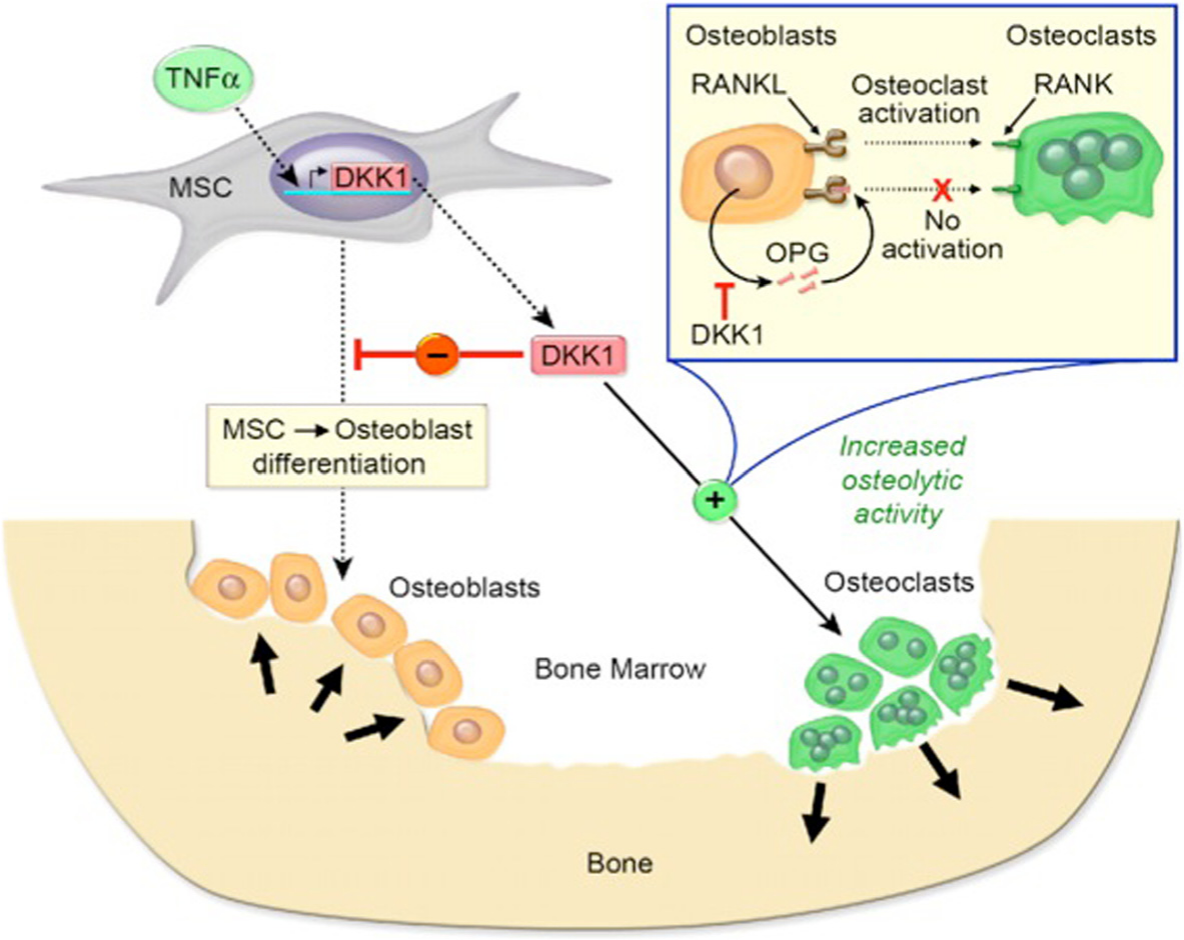
**The role of Dickkopf-1 (Dkk-1) in bone development.** Adapted from Joseph J. Pinzone et al. [6] (with permission). RANKL, a protein expressed on the osteoblast cell membrane, binds to RANK, a receptor located on the osteoclast membrane. RANKL – RANK interaction induces osteoclast differentiation and bone resorption. OPG neutralizes the binding of RANKL to RANK and prevents bone resorption. The Dkk-1 is a soluble inhibitor blocks maturation of osteoblasts and lowers OPG levels, resulting in reduced bone accretion and increased osteolytic activity.

The OPG/RANKL/RANK cytokine axis appears as the final effectors of most of the osteotropic factors already identified. The system consists of the transmembrane protein RANK, its ligand (RANKL), and the soluble receptor OPG. RANKL, a protein expressed on the osteoblast cell membrane, binds to RANK, a receptor located on the osteoclast membrane. This cell-to-cell interaction initiates a cascade of events resulting in activation and differentiation of osteoclasts. OPG is a soluble decoy receptor that binds to RANKL, to limit activation of RANK [12]. Regulation of osteoclastogenesis by osteoblast-derived OPG and RANKL involves Wnt signaling. Evidence from animal models and human studies supports an anabolic role for Wnt signaling in accrual and maintenance of bone mass, mediated by enhanced osteoblast differentiation/activity with concomitant suppression of osteoclast differentiation/activity (Figure 3) [6]. The Wnt pathway is regulated by a large number of antagonists, including the Dickkopf family. The Dkk-1, a secreted protein, is a soluble inhibitor of Wnt, which blocks maturation of osteoblasts [13].

Both pathways, RANKL/RANK/OPG and Wnt, appear to be activated by atherogenic factors [14]. There is a hypothesis according to which calcified aortic valve disease represents an atherosclerosis-like process involving the aortic valve [15,16]. Our study, however, which includes consecutive patients, confirmed previous observations that about half of patients with severe CAS had angiographically normal coronary arteries. Therefore we designed a study which aimed to asses serum RANKL/OPG ratio, Dkk-1 signaling and deposition of calcium in aortic valve in patients with symptomatic CAS in relation to the presence of concomitant coronary atherosclerosis. The study documented significant differences in serum Dkk-1 signaling and deposition of calcium in aortic valves in relation to the presence of concomitant coronary atherosclerosis in patients with symptomatic CAS. Our understanding of mechanisms controlling initiation and progression of aortic valve calcification is limited. Calcification of aortic valve is an actively-regulated multifactorial process. Study results evoke suggestions that concomitant coronary atherosclerosis influences progression of calcified aortic valve disease, or that there are at least two distinct pathogenic entities of aortic valve calcification.

We suggest that the main finding of this study is that elevation of serum Dkk-1 was associated with a significant increase of load of calcium in aortic valve. Rosenhek and colleagues [3] demonstrated that the presence of aortic valve calcium in patients with asymptomatic mild or moderate aortic stenosis was the single most significant predictor of clinical progression. The studied population consisted of consecutive patients who were being considered for aortic valve replacement because of symptomatic CAS. Patients with CAS and normal coronary angiography, with significantly higher Dkk-1 signaling and significantly higher load of calcium were symptomatic at significantly younger age in comparison to patients with CAS and significant coronary atherosclerosis.

Clinical observational studies showed that aortic valve calcification is inversely correlated with low bone tissue mineral density [17,18]. Direct *in vivo* measurements confirmed a paradox of simultaneous osteolysis and ectopic calcification in the same animal [19]. This evidence suggests that osteoporosis may contribute to cardiovascular calcification by adding to a pathological microenvironment that promotes osteogenesis of the aortic valve. During bone resorption, biochemical factors are released into the circulation, contributing to vascular calcification [20]. RANKL – RANK interaction induces osteoclast differentiation and activation, stimulates bone resorption, whereas OPG neutralizes the binding of RANKL to RANK and prevents bone loss. Dkk-1-mediated inhibition of Wnt blocks maturation of osteoblasts. Thereby, Dkk-1 enhances RANKL levels, because osteoblast precursors produce relatively large amounts of RANK ligand [21]. It was confirmed by a clinical studies that serum Dkk-1 expression was highly inversely correlated with bone mass density [22]. It was shown that agents that block bone resorption in animal models also block vascular calcification [23,24]. Dkk-1 antisense oligonucleotide treatment affected bone metabolism by increasing osteoblast numbers and also by reducing RANKL expression, ultimately decreasing osteoclastogenesis [25]. Monoclonal neutralizing anti-Dkk-1 antibody reduces osteolytic bone resorption and increases bone formation in multiple myolema [26]. Our results suggest that it might be interesting for further research examining the potential impact of Dkk-1 antagonists on progression of calcified aortic valve disease.

## Conclusion

Circulating Dkk-1 and calcium deposition in aortic valve differ significantly in relation to the presence of coronary atherosclerosis in patients with symptomatic CAS. A positive association was found between serum Dkk-1 signaling and calcium load in aortic valve in patients with symptomatic CAS and angiographically normal coronary arteries.
